# Student self-reported communication skills, knowledge and confidence across standardised patient, virtual and traditional clinical learning environments

**DOI:** 10.1186/s12909-016-0577-5

**Published:** 2016-02-27

**Authors:** Michelle Quail, Shelley B Brundage, Josh Spitalnick, Peter J Allen, Janet Beilby

**Affiliations:** Curtin University, School of Psychology and Speech Pathology, GPO Box U1987, Perth, WA 6845 Australia; George Washington University, Washington, USA; Citrine Technologies, Atlanta, USA

## Abstract

**Background:**

Advanced communication skills are vital for allied health professionals, yet students often have limited opportunities in which to develop them. The option of increasing clinical placement hours is unsustainable in a climate of constrained budgets, limited placement availability and increasing student numbers. Consequently, many educators are considering the potentials of alternative training methods, such as simulation. Simulations provide safe, repeatable and standardised learning environments in which students can practice a variety of clinical skills. This study investigated students’ self-rated communication skill, knowledge, confidence and empathy across simulated and traditional learning environments.

**Method:**

Undergraduate speech pathology students were randomly allocated to one of three communication partners with whom they engaged conversationally for up to 30 min: a patient in a nursing home (*n* = 21); an elderly trained patient actor (*n* = 22); or a virtual patient (*n* = 19). One week prior to, and again following the conversational interaction, participants completed measures of self-reported communication skill, knowledge and confidence (developed by the authors based on the Four Habit Coding Scheme), as well as the Jefferson Scale of Empathy – Health Professionals (student version).

**Results:**

All three groups reported significantly higher communication knowledge, skills and confidence post-placement (Median *d* = .58), while the degree of change did not vary as a function of group membership (Median *η*^*2*^ < .01). In addition, only students interacting with a nursing home resident reported higher empathy after the placement. Students reported that conversing with the virtual patient was more challenging than conversing with a nursing home patient or actor, and students appeared to derive the same benefit from the experience.

**Conclusions:**

Participants self-reported higher communication skill, knowledge and confidence, though not empathy, following a brief placement in a virtual, standardised or traditional learning environment. The self-reported increases were consistent across the three placement types. It is proposed that the findings from this study provide support for the integration of more sustainable, standardised, virtual patient-based placement models into allied health training programs for the training of communication skills.

**Electronic supplementary material:**

The online version of this article (doi:10.1186/s12909-016-0577-5) contains supplementary material, which is available to authorized users.

## Background

Allied health professionals require high levels of empathy, communication and interpersonal skills in order to carry out their clinical roles efficiently and effectively [1–3]. Such essential skills have demonstrated effects on service quality and patient outcomes [4]. Health professionals are working increasingly within interprofessional teams and in collaborative and consultative models of practice, furthering the need for advanced communication and interaction skills [5, 6]. Colliver et al. [7] suggest that “clinical competence and interpersonal and communication skills are related” (p. 273), whereby generic communication skills underlie the ability to carry out more advanced clinical skills effectively, and the confidence gained from practicing clinical skills further assists in the development of such skills. The evidence for this bidirectional relationship highlights the need for universities to ensure clinical training is targeting both specialised and generic facets of skill development. However, achieving this aim is challenging in the current health and tertiary education climate.

Clinical education in the allied health sciences has traditionally involved the placement of students in community settings with direct supervision and patient/client contact. This model has often been viewed as the clinical education ‘gold standard’ [8]. However, there is a reduction of traditional clinical placements available in the sector due to, amongst other factors, increased student numbers and greater pressures on healthcare systems [8–10]. Tertiary education institutions are under immense pressure to develop strategies that enable students to achieve clinical competency effectively, efficiently, and in an affordable manner. This is in a context where there is limited funding and resources available to support the inclusion of additional clinical placements in allied health curricula. Utilising novel approaches that increase the availability, standardisation and scalability for the teaching of generic skills such as communication, ultimately reduces the burden on the community, and allows training programs to focus on providing the context for specific clinical skills training [11, 12].

### Simulation in clinical education

Simulation provides the potential for sustainable clinical education opportunities, with a focus on specific skills training. However, simulation activities differ significantly in their accessibility, ecological validity, scalability, and affordability.

Changing aspects of clinical placements (such as setting and intensity) does not disadvantage competency development, due to the nature of transferability of skills [9]. Simulated learning environments can allow for greater safety in the learning process but also maximise opportunities for standardisation of experiences within and across cohorts, which is paramount in light of increasing student numbers. Forrest et al. [1] describe the opportunities available for using simulation in training generic skills such as communication and collaboration, particularly when these skills require repeated practice in early stages of training [13]. However, the authors acknowledge the lack of such specific research endeavours in this area to date.

A strong theoretical foundation for the use of simulation is provided by Poore et al. [14] in their mapping of simulated learning onto Kolb’s widely used experiential learning theory [15]. Poore et al. [14] propose that the simulation experience itself defines the first phase of learning, namely the students’ Concrete Experience. Participation in the widely utilised ‘debrief’ component of simulation allows students to be facilitated through the Reflective Observation stage of learning. Then the later post-placement reflection allows for Abstract Conceptualisation where students consider their experience, reflect on it and analyse what could be done differently. Finally the students integrate those skills, experiences and reflections and apply them through Active Experimentation in their future placements and working life [15]. This framework of utilising simulation underpinned by Kolb’s learning theory ensures that the overall experience addresses a range of learning styles with maximal learning potential.

For the purposes of the current study, simulation will focus on the use of Standardised Patients (SPs; e.g., clinical interaction with a trained actor as the patient) and Virtual Learning Environments (VLEs; e.g., clinical interaction in a computer-generated virtual environment with a computer-generated virtual patient) and their potential for training fundamental student communication skills.

#### Standardised Patients (SPs)

Standardised patients (SPs) are human actors who have been trained to respond in specific ways to student clinicians (e.g., following a script, reporting specific symptoms). There are clear benefits to student education with the use of SPs, most transparently the reduction in fear associated with dealing with a ‘real’ patient and the greater ‘safety’ of the experience for all parties [1, 10, 16]. SPs offer a degree of standardisation not previously available through traditional clinical placement models, although total standardisation is difficult and relies heavily on the skills of the individual actors [8]. Studies investigating the accuracy of presentation of standardised patients have identified issues around varying individual styles, the need and importance of training, and the realism of portrayals [17]. The use of SPs is often double the cost of traditional clinical education, with the potential need to employ both SPs and clinical educators over the same time period. There is also the establishment cost of provision of the original training for the SP in the first place [10].

#### Virtual Learning Environments (VLEs)

A Virtual Learning Environment (VLE) is a teaching, training, and learning tool designed to enhance a student’s learning experience by including computers and computer generated applications. VLEs are created to achieve specific learning outcomes [18]. Recent educational innovations in the tertiary sector have seen the introduction of a range of technologies that can foster increased levels of engagement, which is a key factor in student learning outcomes [8, 19, 20].

The last decade has seen an exponential growth in the use of VLEs in the tertiary sector, with training institutions investigating VLEs as a standardised and/or cost-effective alternative to traditional methods [21, 22]. Other perceived benefits include an increased capacity to offer various clinical experiences within one location that has access to various VLE-based environments for teaching a variety of clinical skills, ultimately making it possible to target a range of different learning opportunities in one physical location [22]. In addition, VLEs have the potential to provide globally-standardised training tools and assessments, improving the opportunity for equity and consistency in education and training [19].

Similar to SPs, VLEs can also facilitate learning through repeated practice in a safe learning environment [21, 23]. Unlike SPs however, the level of standardisation of the virtual patient is higher given that the virtual patient is controlled by the educator at the design and/or programming stage and in the delivery and use of the technology [22]. If necessary, the same verbal and non-verbal interactions can be consistently delivered across students’ clinical experiences. VLEs, however, do rely on expertise and support from technology specialists to design and develop the system to be a sophisticated and effective a clinical tool. In addition, they typically require ongoing technological support and maintenance [22]. There are inherent limitations regarding the repertoire of comments and reactions of the virtual patients, in that technology is unable to fully replicate totally, the subtleties and complexities of human communication interactions. VLEs involve a significant financial outlay at the beginning for design and construction costs, but once developed the ongoing costs are confined to technology support and salaried staff to administer the training [22].

Meta-analyses have indicated how the use of virtual patients, either as stand-alone learning opportunities or in conjunction with classroom-based instruction, improves students’ knowledge and skills in the areas of clinical reasoning, ethical decision making and communication [24, 25]. Consorti et al. [24] however, noted the relative lack of studies evaluating virtual patients for specific training of communication skills.

### Evaluating student learning in simulation

Many studies utilising simulation for the development of generic communication skills have measured outcomes using both objective assessments of performance and student self-rating scales [26–28]. In addition, a number of studies have focused exclusively on the students’ self-efficacy, using self-rating scales to measure confidence, teamwork, anxiety, and perceived skill and/or knowledge on simulated placements [5, 12, 29, 30].

There is an increasing need for research that directly compares clinical education models such as alternate simulation models with traditional community-based placements [12]. A few studies have investigated clinical education models with relevance to the teaching of specific clinical skills; comparing traditional and SP models [31] or alternate simulation models [32, 33]. However, when the goal is generic communication skills, the limited research available has compared simulated experiences with classroom teaching as opposed to traditional placement experiences [13, 34]. Zraick et al. [34] objectively measured speech pathology students’ competence in interacting with patients, and found no significant difference between students who received training through didactic lectures and those who received a combination of these and an experience with an SP. A similar study by Zavertnik et al. [13] found that students interacting with SPs demonstrated better communication skills but the difference was not significant overall compared to traditional classroom teaching. Deladisma et al. [35] investigated more complex communication skills using objective measures of students’ nonverbal communication and empathy, when interacting with either a standardised or a virtual patient. Despite the patients in the two conditions having identical scripts, students interacting with the standardised patient scored better overall with higher empathy and more appropriate nonverbal behaviours. The findings of these studies provide favourable results for the use of simulated learning environments for training communication skills, but more direct efficacious comparisons are required between simulation and traditional clinical placement models.

Increased pressure on training programs to ensure high quality clinical experiences in the context of increasing student numbers and shrinking budgets, necessitates that clinical education coordinators be best informed regarding the most advantageous and cost effective training methods. Consequently, this study aimed to address the following research questions: (1) Is there an increase in students’ self-rated communication skill (1a), knowledge (1b), confidence (1c) and empathy (1d) following a communication skills placement in which students interact with an elderly patient in either a traditional clinical placement setting (nursing home), with an SP in a simulation laboratory or a virtual patient in a computer generated office setting, delivered in a University clinic room (VLE)? (2) Are there differences in how students evaluate these three placement models?

## Method

### Participants

Sixty-nine undergraduate speech pathology students completed a communication skills placement as part of a third year core clinical education unit. Each participant was randomly allocated to one of the three placement conditions (nursing home, SP or VLE) by being allocated to a numbered placement block using a random number generator, and an additional round of randomisation for any allocations clashing with existing practicum commitments. Sixty-two of these students consented to participation in the current study, representing a 90 % response rate. Prior to data collection, this study was reviewed and approved by the Curtin University Human Research Ethics Committee (approval number PSYCH SP 2014‐35). Prior to the placement, all students enrolled in the unit were provided with an information sheet outlining the nature of the current study. In the information sheet it was emphasised that although participation in the placement and completion of the measures described below were requisite course activities (and to be used as reflective aids), agreement to take part in the current research was (a) entirely voluntary, and (b) their anonymity was assured with de-identification of information to any staff involved in assessment or teaching of the unit. Students indicated consent (or lack thereof) online prior to completing the first measure. Only the data from students who gave consent were included in the analyses.

### Clinical education models

In the nursing home condition, students attended a metropolitan aged care nursing home facility and interacted with a nursing home resident. Residents were chosen by the nursing home director. The clinical educator accompanied the student into the resident’s room, but only participated in the interaction if it was deemed important and appropriate to do so (for example, if a student was unable to respond appropriately to personal information disclosed by the resident).

The SP was a trained elderly male actor who was experienced in allied health student training. The student interacted with the SP in a simulated nursing home suite in a University simulation laboratory. Both the actor and nursing home residents in the traditional placement condition were advised by the clinical educator that the student was meeting with them to have a natural, initial conversation. The actor was also provided with a list of the virtual patient’s pre-programmed responses, and encouraged to replicate the range in supportive and challenging responses.

According to the virtual patient framework created by Kononowicz et al. [36], the virtual patient used in this study can be classified as a conversational character, for the competency dimension of patient communication skills within the category of education. The VLE consisted of a computer-generated virtual patient projected on to a 65-inch high definition flat screen television wall mounted in a clinical training room adjacent to a one-way observation mirror. The virtual environment included a hallway, generic office space, and the virtual patient seated in a wheelchair at a desk. The software also included a user interface that contained all 45 possible responses produced by the virtual patient. These responses were organised into the following categories; *Profile* (demographic information), *Concern* (health and other concerns), *Challenge* (a series of statements to confront or challenge the students), *Affective* (verbal and non-verbal reactions expressing emotion such as crying, slamming the table with fists), *Agree* (statements and nonverbal behaviours allowing the virtual patient to agree with the questions posed), *Disagree* (statements and nonverbal behaviours allowing the virtual patient to disagree with the questions posed), and *Function* (a set of statements about activities that the virtual patient enjoyed). The clinical educator was situated in the adjacent observation room with a view of both the student and the virtual patient. The clinical educator controlled the virtual patient’s verbal and non-verbal responses and reactions via a laptop and viewed the virtual patient via an additional computer monitor linked to the laptop. As with all conditions, students were informed that they were required to have an initial conversation with the elderly male virtual patient to find out about his background, his general interests and any problems that he might be experiencing. One clinical educator oversaw all three placement models over a period of five weeks.

### Measures

One week prior to their assigned placement, and again immediately following, each student received an online questionnaire with a request to complete it within 24 hours. The pre-placement questionnaire contained a consent form followed by a series of demographic questions, measures of self-reported communication skill, knowledge and confidence, and the health professions student version of the Jefferson Scale of Empathy (JSE-HPS, [37]). The post-placement questionnaire contained the skill, knowledge and confidence measures repeated, the JSE-HPS and several qualitative questions pertaining to the placement experience. A copy of the questionnaire items is available in Additional file 1.

#### Self-reported communication skill, knowledge and confidence

The self-rating scales were developed by the authors based on the Four Habits Coding Scheme, a validated instrument used for describing and assessing clinician communication skills [38, 39]. The student self-report measures indicated (a) how certain they were that they could complete the task specified (skill); (b) the extent of their practical and/or theoretical knowledge (knowledge); and (c) their confidence regarding their ability to perform (confidence) nine key communicative tasks on numeric scales ranging from 1 (‘very uncertain’, ‘not at all knowledgeable’, or ‘not at all confident’, respectively) to 7 (‘very certain’, ‘very knowledgeable’, or ‘very confident’, respectively). Cronbach’s alphas calculated for pre-/post-placement skill, knowledge and confidence were .93/.92, .92/.92 and .93/.92 respectively, indicating that each measure was internally consistent. Responses to the nine self-report items on each measure (Appendix A) were summed to provide an overall score for each construct.

#### Jefferson scale of empathy – health professions student version

The JSE-HPS is a 20-item scale designed to index an “orientation or behavioral tendency toward empathic engagement in patient care” ([40], p. 289). The scale includes items such as “patients feel better when their health care providers understand their feelings” and “attention to patients’ emotions is not important in patient interview” (reversed). The student responds to each item on a 7-point numeric scale ranging from 1 (‘strongly disagree’) to 7 (‘strongly agree’), and after the 10 negatively worded items have been reverse coded, item responses are summed to provide an overall empathy score. Fields et al. [40] report adequate internal consistency (α = .78) and test-retest reliability (*r* = .58 and .69 for three and six months respectively) for the JSE-HPS. In the current study, Cronbach’s alpha was .82 at pre-placement and .87 at post-placement.

#### Placement evaluation measures

Following the placement, the students indicated the extent to which they agreed (on a scale from 1 = ‘strongly disagree’ to 7 = ‘strongly agree’) with 12 statements regarding their impressions/evaluation of their assigned placement. Cronbach’s alpha was .89, indicating internal consistency. After reverse coding one item, responses were summed to provide a global index of how favourably the placement was evaluated.

Finally, the students completed four open-ended questions regarding perceived benefits and challenges of the placement and suggestions for improving the overall clinical experience. The answers to the questions were imported into NVivo 10, and subjected to thematic analysis following the procedures described by Braun and Clarke [41].

### Procedure

Students were randomly allocated to one of the three placement models using placement allocation software, and allocated to one four-hour placement slot that fell within a five-week period. One week prior to the placements commencing, a brief information session was provided, including the expectations of the placement and suggestions and guidance for interaction skills for building rapport with patients. During this session students were informed of the placement model they would be completing. Students received their pre-placement questionnaire one week prior to their scheduled placement slot and received their post-placement questionnaire immediately following their placement.

Students were informed that the aims of each placement were consistent and that each would involve a small group induction with the clinical educator, an individual 30-min interaction with their communication partner and a small group debrief session. After each individual interaction, students were given immediate feedback regarding their interaction exchange by their clinical educator. The small group (up to six students and one clinical educator) debrief session utilised facilitative questions to support student reflections [42].

For all three placement conditions, students were informed that clinical and emotional support for the interaction would be provided by the clinical educator upon request. Students were given permission to leave or cease interactions at any point in the session. Once all students completed their clinical placements, a whole class tutorial was conducted to provide a forum for the students to share and reflect on their experiences. For equity purposes, students were offered the opportunity to participate in the other clinical placements models after completing their assigned placement.

## Results

Participants in the nursing home condition (*n* = 21; *M* age = 21.05, *SD* = 2.04; 100 % female) did not significantly differ from those in the SP (*n* = 22; *M* age = 23.5, *SD* = 6.28; 95 % female) or VLE (*n* = 19; *M* age = 23.53, *SD* = 7.29; 100 % female) conditions in terms of age, *F*(2, 58) = 1.27, *p* = .289, *η*^2^ = .04, or gender distribution, *χ*^2^ (2, *N* = 62) = 1.85, *p* = .397, ϕ = .17. The majority of all participants (80 %) reported that they had interacted with older adults as part of a previous observational placement within their course; as well as with older family members outside of the course (90 %). Condition was not significantly related to students’ likelihood of having interacted with an older adult either within, *χ*^2^ (2, *N* = 62) = 5.18, *p* = .075, ϕ = .29, or outside of their course, *χ*^2^ (2, *N* = 62) = 1.40, *p* = .496, ϕ = .15.

To address research question 1, four, one-tailed paired-samples *t*-tests were used to compare students’ self-reported pre- and post-placement communication skills, knowledge, confidence and empathy following a communication skills placement. To protect against the inflated risk of making Type 1 errors when conducting multiple comparisons, an alpha level of .0125 was used for each. As illustrated in Table 1, all three groups of students self-reported significantly higher post-placement communication skills, knowledge and confidence (Median *d* = .58). However, only members of the nursing home condition self-reported higher post-placement empathy.

**Table 1:** Summary of differences between pre-placement and post-placement scores on the four outcome variables for each group of participants

	Nursing Home (*n* = 21)				SP (*n* = 22)				VLE (*n* = 18)			
	Pre- *M(SD)*	Post- *M(SD)*	95 % CI Diff	*d*	Pre- *M(SD)*	Post- *M(SD)*	95 % CI Diff	*d*	Pre- *M(SD)*	Post- *M(SD)*	95 % CI Diff	*d*
Skill	4.86(1.13)	5.44(0.88)	[0.27, 0.91]**	.59	4.11(1.08)	4.59(1.00)	[0.06, 0.91]*	.47	4.32(0.79)	4.77(0.78)	[0.10, 0.80]*	.57
Knowledge	4.68(1.01)	5.30(0.82)	[0.23, 1.02]*	.69	3.92(1.02)	4.59(1.01)	[0.28, 1.05]**	.65	4.15(0.74)	4.63(0.65)	[0.13, 0.84]*	.70
Confidence	4.69(1.15)	5.26(1.00)	[0.17, 0.95]*	.52	3.86(1.01)	4.60(1.13)	[0.21, 1.26]*	.69	4.19(0.79)	4.62(0.73)	[0.08, 0.78]*	.57
Empathy	5.80(0.50)	5.99(0.47)	[0.07, 0.32]*	.40	5.55(0.67)	5.44(0.88)	[-0.50, 0.28]	-.14	5.84(0.33)	5.79(0.36)	[-.017, 0.08]	-.14

Note: 95 % CI Diff = 95 % confidence interval for the difference between two related means. *d* = Cohen’s *d* for the standardised difference between two related means. * *p* < .0125, 1-tailed; ** *p* < .001, 1-tailed

To determine whether or not the degree of pre- to post-placement change varied as a function of condition, a series of between-groups ANOVAs (again with α = .0125) were conducted. The three conditions did not significantly differ in terms of pre- to post-placement change in skill, *F*(2, 59) = 0.25, *p* = .780, *η*^2^ = .01, knowledge, *F*(2, 59) = 0.16, *p* = .851, *η*^2^ = .01, confidence, *F*(2, 59) = 0.52, *p* = .596, *η*^2^ = .02, or empathy, Welch’s robust *F*(2, 36.22) = 4.31, *p* = .021, *η*^2^ = .05. With the exception of empathy, these effect sizes would all be characterised as ‘small’, according to Cohen’s [43] conventions.

To address the second research question, whether there are differences in how students evaluate the three placement models, students’ global evaluations of each placement were compared using a between-groups ANOVA (with α = .05), which was statistically significant, *F* (2, 58) = 6.27, *p* = .003, *η*^*2*^ = .178. Follow-up post-hoc comparisons using Tukey’s HSD indicated that the nursing home condition participants (*M* = 4.97, *SD* = 0.98) evaluated their placement more favourably than the VLE condition participants (*M* = 3.77, *SD* = 1.08; *d* = .89), but not more favourably than the SP condition participants (*M* = 4.69, *SD* = 1.23; *d* = .21). Furthermore, the SP placement was evaluated more favourably than the VLE placement (*d* = .79). To identify the possible source of these differences, each of the 12 evaluation items was analysed separately. Due to the exploratory nature of these analyses, each ANOVA and each follow-up post-hoc comparison (using Tukey’s HSD, where applicable) was evaluated for significance at an alpha level of .05. Consequently, they should be interpreted with the appropriate degree of caution. As can be seen in Table 2, the VLE participants found the placement experience significantly less realistic and natural than the SP participants, who found their placement significantly less realistic and natural than the nursing home participants. Compared to the other two groups, the VLE participants also found the experience less consistent with real world experiences, and reported lower levels of engagement and enjoyment. Furthermore, both simulation groups recalled a greater level of anxiety leading into the placement than the nursing home participants. However, the three groups did not differ in terms of their perceptions regarding the amount of learning they derived from the placement, the helpfulness of the placement, its usefulness for learning how to interact with real patients, their degree of skill improvement, and the value they derived from the clinical educator. All quantitative data is available in Additional file 2.

**Table 2:** Summary of differences between item level evaluations of the three placement conditions

	Nursing Home (*n* = 21)		SP (*n* = 22)		VLE (*n* = 18)		
	*M*	*(SD)*	*M*	*(SD)*	*M*	*(SD)*	*F*
The experience was similar to experiences I will encounter clinically.	4.30	(1.92)	4.45	(1.53)^c^	3.21	(1.40)^b^	3.40*
The experience was realistic.	6.20	(1.06)^b,c^	4.64	(1.56)^a,c^	2.42	(1.02)^a,b^	44.72*
I did not learn much by participating in this experience.	3.55	(1.90)	3.45	(1.92)	3.53	(1.50)	0.02
I enjoyed this learning experience.	5.42	(1.54)^c^	5.14	(1.55)^c^	3.89	(1.66)^a,b^	5.05*
The experience helped me to learn how to interact with real patients.	4.95	(1.54)	4.27	(1.70)	3.79	(1.62)	2.53
My skills have improved after participating in this experience.	3.90	(1.45)	3.68	(1.67)	4.05	(1.68)	0.28
The clinical educator facilitated my learning in this experience.	5.50	(1.10)	5.27	(1.70)	6.11	(1.15)	2.00
The interactions with the older adult seemed natural.	6.50	(0.61)^b,c^	5.09	(1.74)^a,c^	2.21	(1.18)^a,b^	56.14*
I was anxious prior to participating in this experience.	3.30	(1.84)^b,c^	5.14	(1.86)^a^	5.26	(1.45)^a^	8.05*
The experience was consistent with real world experiences.	5.50	(1.64)^c^	4.45	(1.63)^c^	3.16	(1.50)^a,b^	10.57*
I felt a high level of engagement during the experience.	5.25	(1.45)^c^	5.05	(1.65)^c^	2.58	(1.61)^a,b^	17.49*
Overall, the experience was helpful in my learning.	4.35	(1.93)	4.50	(1.74)	4.05	(1.61)	0.33

^a^ pairwise comparison with nursing home condition significant at *p* < .05. ^b^ pairwise comparison with SP condition significant at *p* < .05. ^c^ pairwise comparison with VLE condition significant at *p* < .05. * *p* < .05

With regards to research question two, the students’ evaluation of the placement models, students’ answers to the four open ended questions revealed the following themes: *Challenge, Realism and Authenticity, Clinical Value of the Experience, Value of the Clinical Educator, Absence of Context,* and *Physical Environment.* These will be briefly outlined and illustrated in the paragraphs that follow. A full summary of responses is available in Additional file 3.

The first theme to emerge from the qualitative, open-ended responses was *Challenge*, which was manifest in the majority of the student responses. The VLE (referred to as the ‘avatar’ by participants) was reported as more challenging in comparison to the other two conditions. Interestingly, most of the reported challenges were deemed positive, and promoted reflection and professional development.

*Some of the responses that were made by the avatar were challenging, which allowed me to think about and practice my response, which will help when I actually interact with real adult clients. The challenge of it was an advantage I guess. (VLE)*

However, this example illustrates otherwise:

*The avatar was limited to the answers he could give and … made the conversation hard to keep going. (VLE)*

In contrast, the majority of students in the nursing home and SP conditions found the experience relatively straightforward:

*I found that I was not challenged that much with the resident I was assigned to. I probably would’ve learnt more from a resident who presented with a more severe cognitive impairment or illness. (Nursing home)*

*He was super easy to talk to, initiated conversations and maintained them. While this was lovely, it wasn’t very challenging. (SP)*

Although this sentiment was not universal:

*The person I talked to brought up some controversial issues* e.g. *racism, which meant that I needed to decide how to respond to these statements to avoid conflict. (Nursing home)*

*The client challenged me by being somewhat difficult such as getting frustrated and emotional about being in the hospital. I had never had to deal with that before. (SP)*

*Realism and Authenticity* were also salient issues for a large number of students in all three conditions. The degree of perceived reality varied as a function of clinical placement. In the VLE, the overwhelming consensus was that the experience was not realistic, and that this was deemed unfavourable to the overall experience:

*I could begin to predict what he was going to say and this felt unnatural and advances in a conversation did not seem to go anywhere. (VLE)*

Conversely, the reality of the nursing home experience was seen as a major advantage:

*It was great being given the opportunity to interact with a real person. (Nursing home)*

Contrasting opinions were expressed regarding the authenticity of the SP. For the majority of the student cohort, given that the interaction was with an SP (and not a ‘real’ patient) detracted from the overall experience:

*The patient wasn’t real - and knowing it made this experience seem somewhat pointless. (SP)*

The *Physical Environment* was particularly salient in the reflections of the VLE group. Members of the other two groups who did reference this theme reported that the presence of a physical context enhanced the placement experience:

*It is nice to get an orientation and feel of the place before jumping straight in with assessment and treatment goals. (Nursing home)*

By contrast, the VLE participants reported that the physical space was not appealing for them as a potential therapist:

*Not being able to physically be there with the person, for example pass them water or grab their hand if they are upset and need support, really made it difficult to build rapport with the client. (VLE)*

Many students spoke of the *Clinical Value of the Experience,* in terms of the learning opportunities it provided and how these would help prepare them for future placements and professional practice. These sentiments were expressed by all three groups of students:

*Being given the opportunity to practice and experience what a challenging conversation with an elderly client would be like to help us prepare for real-life situations and think about how we would respond in certain situations appropriately. (VLE)*

However, not all students felt that the experience was valuable:

*It felt like a waste of everyone’s time…. Speaking to an actor for 15 min did not make me better at communicating with older adults. (SP)*

Whilst the value of the overall experience was salient for all three groups, the *Value of the Clinical Educator* specifically was far more salient for the students in the SP and VLE groups, than for those in the nursing home scenario. The majority of references to the clinical educator were positive:

*The supervisor was very supportive and gave us some very useful constructive feedback once we completed the virtual simulation. (VLE)*

A smaller number of students, from both the nursing home and SP (but not VLE) groups, noted that they gained value from peer reflection and would have liked more opportunities for peer learning throughout the project:

*I think seeing how other people handle situations provides valuable learning that could have been used perhaps afterward to aid reflection (and learning) (SP)*

A number of students had difficulty with the *Absence of Context* for their placement (e.g., the absence of case histories or any purpose for the ‘consultation’) and the idea that the opportunity was for generic communication skills development, rather than the advancement of specialised clinical skills. This theme was most prevalent in the responses from the SP and VLE groups:

*Give the students a list of case history they need to find out, otherwise there is not a lot of point talking to the patient. (SP)*

## Discussion

This study compared two simulated clinical education experiences (interaction with an SP in a simulated setting and a virtual patient in a VLE) with a traditional clinical education model (interaction with a community nursing home resident). The outcomes were measured using the difference between students’ self-reported communication knowledge, skills, confidence and empathy before and after the placement, in addition to the students’ satisfaction and feedback on the experience.

### Changes in communication self-efficacy

The findings of this study revealed that students in the three placement models, as a result of their clinical learning experience, all perceived an increase in their communication knowledge, skills and confidence, and that these increases were essentially equivalent for the three different placements. The significant difference found between pre and post placement ratings provides strong evidence that a single communication training experience can bring about positive changes in a student’s communication self-efficacy. As communication skills are integral to practice across the allied health sector [3, 22], a focus on teaching positive communication skills prior to entering community clinical placements has the potential to improve overall placement performance, and ultimately contribute to reducing the burden on the health training workforce. The results in the simulation conditions are consistent with those in the literature demonstrating that simulated learning experiences can increase students’ self-efficacy overall [5, 12, 29, 30]. The measures were subjective perceptual ratings targeting the competency of communication, and the students’ perceived confidence greatly affects their communication competence in the community [11].

A significant improvement in self-reported empathy was also found post-placement, but was limited to the traditional nursing home placement model. This may be a consequence of the nature of the discourse exchanges in the nursing home interaction compared to the simulated placements. Fields et al. [40] include in their definition of empathy the ‘intention to provide help’ (p. 288). Given that there is more likelihood of emotive topics being raised by actual patients, it may be that the students felt more likely to be of assistance in this condition. Anecdotal support was provided by the clinical educator who reported that conversations tended to be longer in duration in the nursing home condition, providing more time for rapport building and exploration of conversation topics, as the underpinnings for the development of empathy.

### Comparison between conditions

The results from this study demonstrated that students in both simulated conditions (SP and VLE) reported similar increases in self-perceived communication skill, knowledge and confidence as did students in the traditional placement condition, but also a similar amount of learning overall and positive, helpful reflections on the experience. This equivalency of learning substantiates the results reported by Sheepway et al. [9] whereby the same competency outcomes are achieved regardless of placement setting. This current study adds in the important cross-modal comparison. Parker et al. [31] reported similar consistent findings between traditional and simulated teaching methods, providing further support that simulation can fill the gap where traditional clinical placements cannot fill the need, and simulated learning can offer similar, and in some circumstances superior, outcomes [44].

A comparison of the two simulation conditions indicated equivalent support for the students’ self-rated confidence, skill and knowledge development. Such comparable outcomes have also been demonstrated by previous research [32, 33] although objective measures were used in both studies. Such findings support future opportunities for training programs to select different simulation models that best meet their financial, logistical and contextual limitations.

### Students’ evaluation of the placement

The student evaluations indicated that the emphasis on generic skills rather than a specific clinical scenario was challenging. Despite the learning outcomes of the placement being centred on student communication and interpersonal skills as generic competencies, students found it difficult to see clear benefit in the absence of clinically articulated goals. The value of the clinical educator in the experience as described by the students reinforces a consistent finding that simulated learning environments still require the ongoing support of a clinical educator to guide the students’ learning through feedback and debrief [1, 5]. A review of the effectiveness of simulation by Laschinger et al. [45] concluded that the value of simulated learning methods is in their role alongside ‘real life’ clinical practice, such is in preparation or a remediation strategy for marginal students, and not as a replacement for traditional learning approaches.

#### Traditional placement setting

The students who underwent training in the traditional clinical model evaluated this experience more positively than did the students who participated in the VLE with the virtual patient, despite comparable pre-post results on quantitative self-report measures. Although not a direct comparison, a report by Newby et al. [22] details students’ lower satisfaction with virtual learning environments, and describes similar student concerns regarding the perceived restrictions of the technology. The traditional placement was viewed as intrinsically rewarding for students, as they reported positive experiences with a nursing home resident, who they perceived as also benefitting from the interactions. This reciprocity of benefit corresponds with the self-reported increases in students’ ability to convey empathy with the nursing home residents, compared to students in the simulation learning environments. However, in terms of competency development, the students described how the interactions did not challenge them as much given that they didn’t need to repair communication breakdowns for example. It is such fundamental skills that will prove most beneficial not only in future community placements but in future workforce endeavours as allied health professionals [1–3]. Ultimately, it is the traditional model that is the most unsustainable with increasing numbers of students and finite facilities. In addition, the variation in placement and supervision quality between placement sites and clinical educators cannot be controlled for, compounding potential inequities in student experiences.

#### Simulated placement settings

The finding that students in both the SP and VLE conditions reported being more anxious prior to the experience than did students in the traditional placement model, contrasts with research describing the use of these simulated learning tools as a method to reduce student anxiety in non-threatening environments [10, 21]. Given this was the first time that SPs and VLEs were used in this course, it is possible that the participants were confronted by the novelty of simulation [1], having only experienced traditional clinical placements until this time. As students knew their placement condition prior to completing the pre-placement survey, this timing may have then biased their responses, particularly if they didn’t receive their ‘preferred’ allocation. The comparison between SP and virtual conditions by Deladisma et al. [35] found a significant, although weak, correlation between objective ratings of student anxiety and empathy (where higher scores represent both reduced anxiety and greater empathy). Given the anxiety reported by students in the SP and VLE conditions in this study, this may also explain the significant difference in self-reported empathy found only in the traditional placement condition.

The qualitative data revealed a perception that both simulated learning environments were viewed by students as inferior clinical education models. In the light of the demonstrated benefits for communication competency in simulated learning environments including results from this study [1, 10, 16, 24], there is a need to address student preconceptions regarding simulation-based clinical education models, and reframe ‘challenging’ experiences as positive learning opportunities.

Benefits of simulated learning include the opportunity for consistent or individualised delivery and repetition of practice [25]. Both the SP and VLE placement conditions offer these benefits, although on a larger scale when all students are completing the one placement type, consistency would be harder to maintain in the SP condition. Hill et al. [17] describe the need for ongoing training of standardised patients which has a financial and logistical impact on the sustainability of the model. The design and programming of VLEs however ensures consistency can be maintained, in addition to offering individualised experiences for each student as required. Clinical educators can decide online how challenging to make the experience for each student. Such programming may limit this model for use with more complex clinical skills training (such as those involving physical touch or in depth therapeutic procedures), but for generic skills such as communication, it offers the greatest level of standardisation and/or online flexibility. In addition, it has the potential for repeated practice across a range of different virtual settings from the one physical location. With the future incorporation of voice recognition technology (see [22]) there is an opportunity for the VLE model to have the greatest geographical reach, with unlimited repetitive practise.

### Limitations and future research

This study used a between subjects design which meant that no student completed all placements, and that the placements were inherently different in a number of ways. As a result, the conditions were not equivalent. All students in this cohort completed one of the three placements (regardless of their consent to participate in the study), and it formed an essential part of their preparation for final year adult placements. Clinical education was therefore the priority, and so each condition was designed to most naturally reflect how the placement might be conducted in the future. This resulted in some inconsistencies between conditions in terms of the conversational control (e.g. the lack of restrictions in the nursing home interaction, provision of direction but not exact scripting for the SP, and flexible use of a limited set of responses in the VLE condition), clinical educator involvement (e.g. the clinical educator was occasionally engaged in conversation by the resident directly in the nursing home) and the length of interactions. Future research is warranted to investigate within subject evaluations of the three placement models, in addition to more rigorous matching between conditions.

In terms of the structure of each placement, the participants in this study completed a standard, single interaction of up to 30 min in length. It would be of future benefit to investigate whether increasing the length and/or number of interactions within each placement model influences the perceived outcomes. The opportunity to obtain feedback and engage in repetitive practice are factors highlighted as essential to simulated clinical education in order for students to implement changes in their subsequent interactions and maximise the learning opportunities available [1, 22]. Increasing the number of output responses the virtual patient can produce (as controlled by the clinical educator) would also assist the perceived limited realism and minimise the predictability of responses that were articulated by students in the VLE condition.

The students’ comments that the focus on communication and not specific clinical skills in this placement made it challenging may reflect the timing of the placement positioned during their active clinical placements. According to the Zone of Proximal Development concept (as described by Lev Vygotsky) when considering tasks and users in simulation, activities should ideally fall just beyond students’ comfort zone to aid optimal learning [1]. In reality, it may be that the participants in this study had passed the zone of development where this placement was most useful, requiring either more clinically challenging content or for the placement to be directed towards an earlier student cohort more consistent within their zone of proximal development.

The small sample size restricted examination of the factor structure of the self-report and evaluation measures [46] and thus use of a larger sample is recommended in future.

This study aligned with other research regarding student self-reported measurements of simulation experiences for clinical training [12, 29–31, 44, 47, 48]. Future research could incorporate objective outcome measures of students’ communication competence, and assist in illustrating to all stakeholders (including the students themselves) whether the models are objectively comparable. Correlation of self-reported measures with long term clinical skill evaluation is also recommended to investigate the final Active Experimentation phase of learning [15], and measuring any long term beneficial integrated learning from the placement.

## Conclusion

Students reported significantly higher communication knowledge, skill, and confidence after completing a conversational interaction with a communication partner, regardless of whether this occurred with a standardised patient, virtual patient or nursing home resident. In addition, students described the simulated placement conditions as being more challenging, and emphasised the importance of the clinical educator in the learning experience.

This study adds to both simulation and tertiary health education literature through the unique, direct comparison of students’ communication self-efficacy across simulated and traditional clinical placement models. Given the comparable learning outcomes obtained across the three different clinical models in this study, the value of the technological applications offered by VLEs have demonstrated future potential for communication skills training in the education of allied health students. Of particular relevance in the light of reduced resources for sustaining traditional models are the standardised training opportunities for feasible, efficient and effective clinical education models for the future.

### Availability of data and materials

Questionnaire items used are detailed in the attached document. The raw qualitative and quantitative data from the analyses used in the study are also available in the attached files. Demographic and prior experience variables have been removed from the quantitative data file to ensure the confidentiality of participants and clinical educators. For similar reasons, minor redactions and edits have been made to the qualitative data.

## Additional files

Additional file 1:
**Questionnaire items.** Questions provided to students pre (self-report items only) and post placement (all items). (DOCX 15 kb) Additional file 2:
**Quantitative data.** Includes all data used for statistical analysis. Demographic and prior experience variables have been removed from the data file to ensure the confidentiality of participants and clinical educators. (XLS 79 kb) Additional file 3:
**Qualitative data.** Responses to open ended post-placement questionnaire items used in qualitative analyses. Minor redactions and edits have been made to ensure the confidentiality of participants and clinical educators. (PDF 126 kb)

## Abbreviations

SP: Standardised patient
VLE: Virtual learning environment
JSE-HPS: Jefferson Scale of Empathy Health provider-student version
