# Supplementary material for: Student self-reported communication skills, knowledge and confidence across standardised patient, virtual and traditional clinical learning environments
Source: BMC Med Educ. 2016 Feb 27;16:73. doi: 10.1186/s12909-016-0577-5 (PMC4769506; doi:10.1186/s12909-016-0577-5)
Supplement: Additional file 1: — Questionnaire items. Questions provided to students pre (self-report items only) and post placement (all items). (DOCX 15 kb) [file 12909_2016_577_MOESM1_ESM.docx]

Items rated for self-report measures (competence, knowledge and confidence)

1. Initiate a conversation with a client regarding their concerns.
2. Obtain a list of a client’s primary concerns.
3. Establish rapport with a client.
4. Assess symptoms of aphasia (or other communication disorder).
5. Explain your professional role to a client.
6. Cope with a situation in which a client disagrees with you.
7. Cope with a situation in which a client reacts emotionally to the information you have presented.
8. Determine if referrals to other healthcare professionals are needed, with supporting rationale.
9. Conclude a consultation by summarizing what was said and where to go next

Items rated for placement evaluation

1. The experience was similar to experiences I will encounter clinically.
2. The experience was realistic.
3. I did not learn much by participating in this experience.
4. I enjoyed this learning experience.
5. The experience helped me to learn how to interact with real patients.
6. My skills have improved after participating in this experience.
7. The clinical educator facilitated my learning in this experience.
8. The interactions with the older adult seemed natural.
9. I was anxious prior to participating in this experience.
10. The experience was consistent with real world experiences.
11. I felt a high level of engagement during the experience.
12. Overall, the experience was helpful in my learning.

Items for additional student feedback

1. Do you feel as though you made the most of this experience? Y/N
    If “N”, please explain what prevented you from doing so…

If “Y”, please explain what aspects were most facilitative to your learning…

1. In your opinion, what were the disadvantages of your experience on this placement?
2. Do you feel the experience in this clinical experience could be improved? Y/N
    If “Y”, please provide some examples where you feel improvements could be made…
3. In your opinion, what were the advantages of your experience on this placement?
