# Supplementary material for: Student self-reported communication skills, knowledge and confidence across standardised patient, virtual and traditional clinical learning environments
Source: BMC Med Educ. 2016 Feb 27;16:73. doi: 10.1186/s12909-016-0577-5 (PMC4769506; doi:10.1186/s12909-016-0577-5)
Supplement: Additional file 3: — Qualitative data. Responses to open ended post-placement questionnaire items used in qualitative analyses. Minor redactions and edits have been made to ensure the confidentiality of participants and clinical educators. (PDF 126 kb) [file 12909_2016_577_MOESM3_ESM.pdf]

Do you feel as though you made the most of this experience? Y/N

If "N", please explain what prevented you from doing so…

Nursing Home:

1. Higher level of previous experience than the placement provided.

Actor:

1. I wish I hadn't known prior that I was speaking with an actor. It made the experience feel more forced and I was very aware that it wasn't a real scenario.

VLE:

1. I feel because I knew I wasn't seeing a real person, it made the experience very different. The environment where the conversation was conducted made it very unrealistic and uncomfortable to communicate in. I felt as though I did not communicate efficienty with the client because of these factors.
2. Lack of briefing (I had no idea what to expect)   No idea where the 'experience' was taking place. why I was seeing Jim, and no background or plan to go from
3. The Learning experience was explicitly described as 'not being an assessment', yet the environment under which we completed the experience felt as though it was a test/assessment, when it should feel as though its just natural conversation.

If "Y", please explain what aspects were most facilitative to your learning…

Nursing Home:

1. Actually conversing with the communication partner and finding out more about nursing homes.
2. Being able to talk to older adults in a non threatening or pressured way
3. Boosting my self-confidence in my abilities to easily communicate with the older adult population
4. Clinical educator facilitated learning
5. For me personally, I work in a [WORKPLACE] and I'm constantly dealing with the general public, majority of which is the elderly so I do feel as though I am more than capable to interact with them. However having said that, talking with people living in a nursing home I found was quite different because there are mixed emotions; my lady unfortunately got upset during our talk and as horrible as it was to see her like that, it gave me the opportunity to learn to deal with the situation which of course is something that will happen in the future.
6. Having a real client made it more naturalistic
7. Having a real life person to talk to
8. Having that opportunity to go in and introduce myself as a speech pathologist, as well as being able to have that almost break in conversation to realise that often the client will fill this gap.
9. Having the conversation with the nursing home patient
10. Having the opportunity to observe a client with mild dementia and to easily build rapport with my client.
11. Having the opportunity to speak to a real resident in a nursing home. This allowed me to identify which strategies were and were not effective for communication.
12. Having the opportunity to step foot into the residential home environment in the speech pathologist role. This is something we did not get the opportunity to do for school placement. It is nice to get an orientation and feel of the place before jumping straight in with assessment and treatment goals.
13. interacting with a real person

14. It was great being given the opportunity to interact with a real person, and the fact that she had mild dementia made it slightly more reflective of possible communication problems I will encounter with people who have communication difficulties
15. natural conversation with a resident
16. The elderly were enthusiastic and keen
17. The naturalistic setting in the aged care facility.
18. The real life experience of a nursing home. For example, seeing their day to day life, where they eat, sitting in their rooms, socialising with their peers and carers.
19. The unproductive nature of the conversation and that this is a realistic experience that may come up in our clinical careers.

Actor:

1. discussing it with my peers
2. discussion following the experience with the other students and the CE
3. I dont think this experience offered too much in terms of clinical skills, but i went in with an open mind nonetheless and the one thing i got out of it was to know that you can never really expect anything when it comes to patients.
4. i really wanted to make use of thee placement because we have very little adult experience in 3rd year.
5. Interacting with an older adult helped a lot, if the actor was a young actor I think it would have been much more difficult to have a positive experience.
6. It was quite realistic, thus good experience.
7. Not being told what was going to happen
8. The CE was very supportive to help facilitate our learning and the client was realistic.
9. The clinical educators tips and feedback
10. The clinical setting - requiring walking into a ward and approaching a client in bed; initiating a conversation with someone in that situation is quite different from a real world non-clinical experience. Also having a time limit on a general conversation Increased awareness of needing to have some succinct automatic explanations / phrases to describe what we do and why.
11. the environment was realistic, very supportive and gives me direction for discussion in the future.
12. The one on one interaction with a real person, even though they were an actor, it still mimicked closely a real life interaction
13. The pre-experience lecture from the CE about what types of questions to ask, and how to gain more information from the client
14. The simulation seemed very real. I had to deal with the client getting upset which may not have occurred in a nursing home. Having a client upset means that you have to figure out how to recognise their feelings while still guiding the conversation in a drection that is helpful to assessment.
15. [CLINICAL EDUCATOR] gave us some really good advice, not only for this experience, but also in talking to any patients
16. When the patient was not responding, being difficult, talking about how much he hated being in hospital etc.

VLE:

1. Although I was nervous and did not perform as I would have liked, this has been a positive learning experience for me, because I am now more motivated to get more experience to improve my skills. I feel the debriefing at the end was also very useful for poviding information on how to interact with elderly clients.
2. Clinical educator

3. Clinical educator support
4. Conversing with a client that asked difficult but common questions, and learning how to answer these challenging questions appropriately. e.g. 'How long will I be here for?' 'When can I drive again'. This was quite helpful, because in the real clinical seting, such as a nursing home or outpatient clinic, there will be clients who ask more challenging questions that we do not always know the answer to.
5. Debriefing after the session
6. feedback from supervisor upon completing placement
7. Finding ways to maintain conversation with someone who isn't very responsive. Phrasing questions simply and using a clear voice when speaking.
8. Having the opportunity to engage with an avatar and not having to worry about potentially offending a real human
9. My Clinical Educator was supportive and helpful, and the feedback session afterwards was interesting, especially when she related it to some of her clinical experiences
10. the clinical educator's discussion after the experience was very helpful in understanding how to act in a similar situation
11. The clinicsl educator was great.the whole avatar experience was awful and confidence deflating. Hated it.
12. The debrief was important where our clinical educator informed us of examples on how we could have responded in certain situations and where this would be applied in real life circumstances. Also being faced with a number of challenges that we are likly to experience in the clinical world gave us an opportunity to think and discuss about appropriate ways of responding, which we can take away with us into future placements/ work experiences.
13. The discussion with the clinical educator afterwards
14. The time it took for Jim to respond is something we can come across in clinic. I quickly learnt to allow more time for a response.
15. This experience helped me remember not to expect clients to be easy to communicate and build rapport with and that I have to ensure I am prepared for a client who may not like to talk as much.

In your opinion, what were the disadvantages of your experience on this placement?

Nursing home:

1. Half an hour just wasn't long enough. Also, as helpful as my clinical educator was, I feel like it would have been more beneficial if she did not participate in the one on one interaction with the elderly person as I felt like it took away from the overal experience. I would have preferred it if had just been between me and the elderly person.
2. Having such a short period of communication, and also being just a session where we conversed, as that would not be the only thing that I would be doing if I was actually a health professional and conducting Speech Pathology sessions with the patient.
3. Higher level of previous experience than the placement provided.
4. I didnt feel as though there were any disadvantages, I think the elderly gained a lot from it too - it's nice to just have someone sit and chat with you
5. I feel that the residents didn't really benefit from speaking with students for only a short period of time. Some residents enjoy chatting and telling their life story to anyone who spends a little time to listen to them.
6. I found that I was not challenged that much with the resident I was assigned to. I probably would've learnt more from a resident who presented with a more severe cognitive impairment or illness. I felt like I was more an enabler than a facilitator of coversation as the resident was very conversational.
7. It was only one 15min to 30 min conversation of which we discussed general topics rather than speech related topics therefore i felt that it was not helpful in extending my learning in

speech. I also think that the fact that did not go towards our hours a a speech pathologist made it more of a waste of our afternoon.

8. none
9. not enough time to experience talking to a range of residents/ personality types and not having the experience rerated to speech therapists role directly (e.g. it was a casual 'chat' that didn't really delve into their communication concerns).
10. One conversation for 15 minutes seems beneficial to the residents but not students. There is no room for growth or practice... didn't feel like I gained much at all.
11. Only being able to talk to one person
12. That it was very short
13. That there was not a more diverse population of older adults to communicate with. We only spoke to one adult each, all who were very willing and compliant, which I don't think fully captures the true population of nursing homes.
14. The fact that this placement only involved talking to one elderly client for 15min didn't provide the opportunity to work with clients with different communication styles and different disorders.
15. The limited time frame and that we werent able to talk to a variety of clients.
16. The limited time to interact with the resident. I also have prior aged care nursing experience, which may have disadvantaged my learning, as I do not feel that this placement added to my current clinical skill set.
17. The short time frame, sitting around for 2 hours whilst in the rotation.
18. The time constraints and also the fact that we were limited to one patient only.
19. There is only so much you can learn about talking to adults from a 15 minute conversation. I did not feel that this interaction was beneficial.
20. Time limit and slightly unorganized schedule
21. Was a little short

Actor:

1. He was super easy to talk to, initiated conversations and maintained them. While this was lovely, it wasn't very challenging
2. I don't believe I had any.
3. I feel that the man could have been more difficult to talk to, he was so lovely! I feel that I would have benefited more from a challege. e.g. If the man had aphasia or dysarthria or a hearing impairment.
4. I felt that I wasn't sure of what I was supposed to be getting out of the conversation, which made me confused and very self aware.
5. I find that part of being genuine and sincere in my communication is to talk 'to' the person; I kept trying to speak to the actor rather than the 'patient' in the room - I could see him in there and was responding to him rather than the character he was paying.
6. it was really easy to talk to the patient. i already know how to talk to an adult, and i was underwhelmed by how much skill you actually required to complete the placement. i left knowing 100% i could cope it a normal patient but i did not learn anything bout communicating with patients that i didn't already know.
7. It wasn't as naturalistic as a real person and there could have been better one on one feedback.
8. Knowing it was an actor
9. Knowing that is was an actor made the experience a little less professional than it would be with a patient.
10. Knowing that the client was an actor
11. Knowing that the person was an actor, the fact that it took a long time for a conversation slightly less than 30min.

12. Not being able to view other peoples/students interactions.. I think seeing how other people handle situations provides valuable learning that could have been used perhaps afterward to aid reflection (and learning)
13. Possibly just the fact that we knew he was an actor, although after the first 5 minutes this wasn't a problem
14. The fact that we knew the client in the simulation was just an actor and that may have influenced how we reaction to the situation since we knew it wasn't "real".
15. the idea that the simulation was an actor, sitting in the back of my mind
16. The patient was quite young (60s) and didn't have cognitive/hearing issues. It was just like speaking to any normal adult so it didn't really challenge me at all. Perhaps it was helpful for students just out of high school who don't speak with adults of tat age group but i would have preferred a more challenging patient.
17. the patient wasn't real - and knowing it made this experience seem somewhat pointless. i feel i would have enjoyed this experience more if i was placed in a real nursing home with a real client.
18. The situation seemed reasonably realistic but due to knowledge that it was a simulated experience it seemed less genuine which made it difficult to interact as naturally as possible.
19. We knew the client was an actor which changed the authenticity of the situation Lack of direction - no aim/ goal/ communicative intent

Avatar:

1. I felt awkward interacting with the avatar and I think I would have displayed better clinical skills interacting with a real person. Also my anxiety was increased because I knew I was being filmed.
2. As I assume the avatar only has pre-programmed responses I could begin to predict what he was going to say and this felt unnatural and advances in a conversation did not seem to go anywhere. Also, it was difficult to respond in some cases as we did not hae an idea of the setting or context in which the conversation was taking place (hospital, nursing home, rehabilitation center). I think this could be important to know when the avatar asks us something we do not have the answer for so we know who to seek nformation from to discover the answer from him.
3. Avatar wasn't very realistic.
4. Even though I still learnt through the experience with the avatar, it's not the same as communicating with a real patient.
5. I don't feel like I gained much in terms of my clinical communication skills, due to the fact that the avatar didn't respond positively to any questions I asked him, although I tried a range of open and closed questions, both about his past and his experince in the nursing home currently. He also didn't respond to me talking about myself. Given the fact that nothing I tried elicited a response, other than I don't know, or yes/no answers, I don't know what aspects of my communication were strong or weak duing the interaction. It also felt distinctly unnatural and I felt very uncomfortable, as it was like I was talking to myself for 20 minutes. The lack of feedback during the conversation was the most disappointing aspect.
6. It was not a realistic and natural encounter.
7. It wasn't real. I had no idea where I was meant to be (i.e. nursing home? hospital?) I had no idea how to respond to Jim's continuous "I don't know"s
8. Knowing it wasn't a real person and there wasn't a real background story for the person, also not being given a clear idea about the environment they were in.
9. Not actual person.
10. Not being able to communicate with an actual eldery person

11. Not talking to an actual person. I feel that in a real life experience, there would be more environmental cues and patient knowledge to be able to maintain or continue an engaging conversation.
12. that the avatar was limited to the answers he could give and thus limited made the conversation hard to keep going
13. The avatar said I don't know and I don't remember to almost everything. There were no props to help initiate and lead interactions. The whole experience felt unnatural. There were no facial expressions and minimal body language to help gauge the effetiveness of the interaction. The avatars responses were negative or neutral, there weren't positive responses (other than yes) There was no case history information provided, so when the avatar said things like his head hurt, he was dizzy or asked whenhe would get better, I wasn't able to provide any counselling or reassurance. The positioning (directly opposite, with a table between) was a barrier to communication. This goes against everything we have been taught about building rapport with clients. The client did not provide the longer responses to open ended questions that would be expected. Usually you would be able to get some sort of longer response on a topic.
14. The environment of the conversation (desk, chair and the positioning of the avatar, dark room, no photos of the client or anything meaningful to generate conversation from). This made the experience feel unrealistic.
15. The lack of information provided prior to the actual experience. It would have been helpful to provide some background and specifics about the context the adult was in prior to starting the conversation.
16. the unnatural VLE set up
17. The virtual simulation was a bit unrealistic at times as it was difficult to gage the client's emotions and thoughts.
18. Yes, it was very unrealistic and made me feel a little bit uncomfortable as it was not a real person. With this placement, you do not have any other factors such as body language and facial expressions to help contribute to conversation.

Do you feel the experience in this clinical experience could be improved? Y/N
If "Y", please provide some examples where you feel improvements could be made…

Nursing home:

1. Conversation with clients related to their illness and what strategies they use in everyday life.
2. Done in first year, so people can get an idea of the client's we might work with.
3. Exposure to more than one client
4. I feel like this should be done in first year as it would be more beneficial then. Most students in third year have had experiences talking with older adults - anyone who has grandparents will have had an experience like today's nursing home visit, as mos of the patients were fine and had no communication difficulties - so I feel that in the business of third year, this prac seems a little out of place in terms of importance and would be much better suited to earlier years.
5. If possible access to more clients, perhaps the possiblity to participate in some of their daily activities and the possiblity of seeing clients who were slightly less compliant or uninteligible.
6. Longer interaction with client, CE just on standby vs participating in conversation, would be good to have clients who present with communication difficulties (even if mild difficulties)
7. More diverse population of older adults to communicate with, prhaps with more varying conditions too.
8. More structure and more time. Perhaps more education beforehand.

9. more that one session so that students can practice and improve on theri communication skills
10. More time
11. more time for students to talk to more (range) of residents to prepare us for the diversity we will experience
12. More time with the resident.
13. Provide more opportunities to interact with elderly e.g. more elderly people with shorter time for each conversation The placement should not be so long--> should only have to go in for the duration of the experience then post a comment to [LEARNING MANAGEMENT SYSTEM] about the eperience which the CE can reply to giving feedback.
14. Talking with a variety of older adults with differing health statuses Not waiting four and a half hours for a fifteen minute conversation with an older adult Remove this placement and increase the interaction with clients on the [HOSPITAL] placement
15. The idea is good, however I feel that a one-off 15 minute chat with someone didn't really benefit my learning that much- perhaps this would be a good placement for first or second year students, as it is a nice, relaxed interaction with older adults and itroduction into the nursing home setting. However at this stage, I do not feel that this experience has equipped me with skills needed to interact with older adults with communication impairments
16. Timing,as we had to look for the elderly who were not always in the room
17. Using a range of residents, as the same five residents were used each week.
18. Waiting around for your turn and for feedback was a little bit frustrating. I understand that we were encouraged to do our clinical maps however the resources (power plugs and desk/chairs) were not available.

Actor:

1. By providing the student with an aim/goal of the conversation. Even a small goal would have made me feel I had a purpose of being in the room with him, as opposed to being watched and having a aimless conversation.
2. Giving a case history and a actual problem
3. Have a real resident from the nursing home come and do the simulation or not e told that it will be an actor.
4. Have the students not know the client was an actor
5. Having the actor providing more of a challenge Also I think if this placement continues it would even be beneficial to do it more than once/ with more than one scenario- for example with an actor that pretends to have cognitive difficulties, someone withbehavioural issues and then someone that was as easy to talk to as John was
6. I DID NOT LEARN ANYTHING FROM THIS EXPERIENCE- I'M COMFORTABLE TALKING WITH OLDER ADULTS AND DID NOT FIND THIS EXPERIENCE BENIFICIAL
7. I feel thankful that more opportunities are trying to be made for us, but I honestly think if you don't have enough real life situations, just rule the prac out, i don't think this was a relevant or helpful placement, it felt more like a waste of eveyonestime. I can't imagine the virtual avatar was much better. Speaking to an actor for 15 minutes did not make me better at communicating with older adults.
8. make the conversation more difficult
9. More detailed brief. Ie this guy has been in hospital for a few weeks, nurses notice he is coughing after eating/drinking. You need to collect a case history ect.
10. Older adults with dementia, hearing issues, cognitive issues etc.
11. Reviewing and analysing others interactions as a group with the CE. Open discussion should be facilitated to encourage further learning.
12. the CE would have more time with you individually to go through what you did well and what you could improve on. some corresponding literature make the situation harder, the

patient is more difficult to communicate with. give the students a list of cse history they need to find out, otherwise there is not a lot of point talking to the patient.

13. We were told to just have a chat with them, and that we didn't have any objectives in the conversation. That felt unrealistic and since we haven't had much experience gathering useful information such as case history data I think it would have been good t have done that as well instead of solely building a rapport with someone we knew we wouldn't see again.

Avatar:

1. Clear environmental setting given prior to beginning conversation, so that you can begin to form questions or have an idea as to what kind of situation the client is in. 2. It was confusing when first entering the room and not seeing the patient straiht away. Was unsure when to begin talking to the 'client'
2. A bigger range of responses could be an option
3. A more natural feel to the interaction, and the avatar programmed to respond more
4. A wider range of pre-programmed responses. Placing the client in a particular setting so students can draw off of the environment for conversation opportunities.
5. All students should be involved in this experience
6. Commencing the experience as the participant enters the room would make it more realistic.   Giving the participant a basic case history prior to the session Giving the avatar a bit of a back story so that he can respond with more than just one sentence o open ended questions.
7. Either: - programming more responses for Jim - preparing students on how to react when their client has a limited number of programmed responses
8. Having more than one opportunity to converse with the avatar to get more experience.
9. I think the interaction with the avatar would feel more natural by reducing the distance between the avatar and the student and having the avatar at eye level to the student.
10. I would have liked to go into a nursing home and chat with an actual person
11. It would have been helpful to provide some background and specifics about the context the adult was in prior to starting the conversation.
12. Make the environment more realistic, so that the communication with the client is more realistic. Not being able to physically be there with the person, for example pass them water or grab their hand if they are upset and need support, really made it diffcult to build rapport with the client. It felt more like a test than an experience
13. maybe by adding increasing the number of answers or topics that the avatar could talk about.
14. More realistic, more cues in envitonment.
15. providing a simulation or real life nursing home placement, rather than using a VLE
16. Providing avatar with more responses
17. The avatar needs to be programmed with more responses, so that feedbCk can be made for good communication attempts. Perhaps it could be broken up into five minute conversations, so that feedback could be provided via the Clinical Educator, so that we coul then apply this to the next conversational block. The environment in the room itself was very cold and sterile.

In your opinion, what were the advantages of your experience on this placement?

Nursing Home:

1. A lot of people in our group have had a lot of experience with elderly people. We had in depth discussions about the elderly people we frequently interact with and the conditions they present with. The CE was very informative and helpful befor, during and after the placement.

2. a relaxed introduction into a nursing home setting- I will know generally what to expect when entering a nursing home
3. Actually talking with a older person in the nursing home.
4. Conversing with an elderly patient.
5. Experiencing a nursing home and real life conversation with older patients. Learning to cope with memory loss, word finding difficulties in conversation.
6. Getting to talk to different kinds of people
7. Got to interact with an elderly at a nursing home for the first time and experience what it was actually like to be in a nursing home environment
8. I thought the support from our CE was great, as well as being able to experience what it is like in a nursing home (if people have not experienced this before). I thought it was good exposure to the different smells and how it appears.
9. It is a very good experience for hose who have not had many chances to communicate with oloder adults, and it can help with confidence when communicating with this population on later placements.
10. It was very warming to be able to communicate and establish rapport with an elderly client.
11. more confidence gained in my abilities to interact with older adults professionally yet on a personal level
12. Overall, it was a great experience! It helped me feel more at ease when thinking about dealing with adult clients. It was also a wonderful eye opener to see that older people each have unique stories and rich histories, and that they are generally willingto help our profession
13. Talking to residents at a nursing home is generally going to be unpredictable. You can't always prepare a script for what you are going to talk about.
14. That I got to speak to a real person
15. That the residents were able to get the chance just to have a casual conversation with someone, which for some of them may not happen too often. Also allowed me to briefly get a feel of what it's like to talk to an older adult.
16. The person I talked to bought up some controversial issues e.g. racism, which meant that I needed to decide how to respond to these statements to avoid conflict.
17. the realistic setting of the placement
18. The residents were pleasant
19. Was rewarding to see the resident seem excited to tell me about herself and her family. It did make me start to think about speech rate, volume and clarity when talking to older clients
20. We got to experience the aged care setting. Personally I had never stepped foot in a nursing home so it was a good introduction to it.

Actor:

1. As mentioned above, the actor would get repeatedly upset which may not occur in the nursing home, so having to deal with this the first time I was put into this situation made it so that I could try communicate with the patient and calm them down and thencarry on with the assessment.
2. Being able to interact with a real person - exposure to non verbal language, and being able to experience and shaped by the surrounding environment
3. Being able to practice professionalism skills and rapport building
4. Experience was as realistic as it could be while being simulated
5. gains in confidence.
6. Have experience just chatting to an adult, gets us more prepared for next year
7. I learnt just how important those first few minutes are for both the client and the therapist; the fact that I need to have a number of greetings, statements, explanations and comments

that I am familiar and comfortable with. Also a personal awareness tha I need to have a framework or purpose to work within and towards so as to avoid a randomly expanding and social conversation which I am unlikely to be allowed in the working day!

8. I was expecting to have a superficial conversation with the patient and i was surprised when we had an in depth conversation.
9. it has put me at ease with future adult interactions on placement
10. It was good practice for when we have our adult placements next year. This is very beneficial.
11. Just being a new experience made this placement an advantage, I've never done something like this before so it was rewarding to finish and have positive feedback about how i interacted with the client.
12. learning that you cant come to expect anything before meeting a patient
13. One on one learning and gave me experiences with an elderly client
14. That the interaction was with a person who was presenting a realistic representation of a client.
15. The actor was very good and very friendly. I did really enjoy talking to him and it was a positive experience.
16. THE ACTOR WAS VERY NICE- IT WAS A CALMING ENVIRONMENT
17. The CE facilitated good discussion and consolidated our learning.
18. The fact that the client challenged me by being somewhat difficult such as getting frustrated and emotional about being in the hospital. I had never had to deal with that before so that was something I learned from it.

VLE:

1. Being given the opportunity to practice and experience what a challenging conversation with an elderly client would be like to help us prepare for real-life situations and think about how we would respond in certain situations appropriately. The discussio aspect was also beneficial in doing this to reflect on what things you might say or do differently if faced with the situation again. The animation was also fascinating and the avatar was able to answer promptly.
2. Clinical educator support
3. Gaining a few ideas in the post-experience discussion about what I was meant to be doing during the placement
4. Having the avatar to interact with meant not being afraid of offending the client which was a little relaxing
5. I feel it challenged me and motivated me to get further experience at interacting with elderly people.
6. I thought the actual program itself was amazing, and the avatar responded quickly and naturally to the questions.
7. It has been a challenging and unique learning experience for interacting with an elderly client. I have learnt that I need to be more facilitative in the conversation rather than changing topics when the client is confused or forgetful, it is important toexpand upon what the client has said and provide choices.
8. It has demonstrated the importance of physical interactions (i.e. shaking hands), body language, facial expressions and positioning in building rapport and interacting with the clients.
9. It is a very interesting research idea, and has potential as an effective student learning tool, if improve,nets are made. It was interesting to be involved.
10. Learning how to interact with someone who isn't able to respond to the questions you ask, in the manner you're expecting. Helped me to learn how to re-phrase questions and carry on the conversation.

11. None
12. Not having to worry about actually offending or giving false information to a patient
13. Opens your eyes to some difficulties you could be facing
14. Seeing how an avatar can facilitate learning
15. Some of the responses that were made by the avatar were challenging, which allowed me to think about and practice my response, which will help when I actually interact with real adult clients. The challenge of it was an advantage I guess.
16. The advantages were that I was able to communicate with a more difficult client, who did not elaborate on topics or talk much, which helped me learn what I can do if I end up in this situation.
17. the difficult scenarios experienced in VLE setting are less likely to happen in a nursing home environment, therefore I learnt how to deal with such situations, through supervisor feedback
18. The support from the Clinical Educator.

Nursing Home:

1. I feel that the placement was not long enough and if we had the opportunity to talk to more residents then maybe we would benefit more. One thing I could suggest as an idea is to do more volunteering at aged care homes as part of the course. Maybe doing acertain amount of hours/days at an aged care home over third year. This would allow more learning experiences to communicate with people. Many people in aged care homes enjoy having someone to talk to, so I would prefer if the residents benefit from the cmmunication partners program rather than just focusing on how we learn to communicate.
2. I felt this placement would be more beneficial as a first or second year placement
3. I think that by third year, students are aware of the skills they are lacking and if they feel they have not had a lot of experience with the elderly, that they would value this opportunity. I also had the privilege of doing the avatar the following day nd I learnt a lot more from the virtual learning experience than the residential one. This is due to the range of unexpected emotions and limited responses from the avatar. Although it was more difficult and challenging, I found this more beneficial.
4. It was a good idea to provide us with more clinical experience. The more, the better he experience we receive.
5. It would be great to continue this in subsequent years!
6. Some of us speak to older adults that are not related to us on a weekly basis, so I did not feel that this added anything to my knowledge, it is something I have done hundreds of times before.
7. Was an enjoyable experience however I do not believe it added to my current clinical skills set.
8. Would be more beneficial as a 1st or 2nd year prac

Actor:

1. I don't think this placement is relevant to our clinical skills, I feel [UNIVERSITY] should try to find more than one nursing home to agree to take students and just let students volunteer for a certain amount of time for a few weeks. talking to an actor or eve a real resident for 15 minutes doesnt teach us much.
2. It was a bit frustrating having to wait around for so long, however, I did manage to do work. The warning that we would have down time was good so that we could make the most of it.
3. It was a good experience and i am glad to have participated in this.
4. more one on one feedback about what we did well and what we could improve would be beneficial
5. Thank you for the opportunity :)

6. [CLINICAL EDUCATOR] is lovely!!

VLE:

1. I thought it was still a beneficial experience, because the client's we may see in a real placement may have the same personality and communication difficulties as Jim
2. I was pretty confident about talking to older adults before the placement, but the placement made me anxious and I really doubt my ability to interact with older adults. The discussion brought up many questions, and I have no idea how to answer them, whih furthers my anxiety. I am unsure as to what I did right and wrong, and how to fix my mistakes
3. The supervisor was very supportive and gave us some very useful constructive feedback once we completed the virtual simulation. She gave us some things to consider when working in a real-life older adult setting, and some tips for fostering a supportive ad facilitative interaction.
4. This experience was very different and much more difficult than I expected. This would be a good experience as part of the 2nd year clinical units, to demonstrate the importance of these factors in client interactions. As a third year student, I actually elt that this experience was detrimental. It made me doubt my clinical skills and reduced my confidence in interacting with older people.
5. Was difficult to feel like you were interacting with a real person
